# Fluoroscopy-guided transvenous femoral renal transplant biopsies: a monocentric retrospective study about 109 consecutive patients

**DOI:** 10.1007/s11547-025-02045-4

**Published:** 2025-08-02

**Authors:** Farha Tessier, Charles Roux, Isabelle Brocheriou, Benoit Barrou, Marine Bravetti, Mathilde Aïssaoui, Jean-Charles Bijot, Eloi Varin, Jérome Tourret, Hélène François, Sarah Drouin, Louis Meyblum

**Affiliations:** 1 AP-HP, Hôpital Universitaire Pitié-Salpêtrière, Sorbonne Université, Service de radiologie interventionnelle, Paris, France; 2 AP-HP, Hôpital Universitaire Pitié-Salpêtrière, Sorbonne Université, Service d’anatomie et de cytologie pathologiques, Paris, France; 3 AP-HP, Hôpital Universitaire Pitié-Salpêtrière, Sorbonne Université, Service d’urologie, Paris, France; 4 AP-HP, Hôpital Universitaire Pitié-Salpêtrière, UMR S1155, Sorbonne Université, Service de Transplantation rénale-Néphrologie, Paris, France; 5 Interventional Radiology Department, Hôpital Universitaire Pitié Salpêtrière, 75013 Paris, France

**Keywords:** Image-guided biopsy, Kidney transplantation, Radiology, Interventional, Hemorrhage, Anticoagulants

## Abstract

**Objectives:**

Renal transplant biopsy remains the gold standard for etiological diagnosis of graft dysfunction and subsequent management. When percutaneous renal biopsy (PRB) is contraindicated due to bleeding risk or limited percutaneous access, the fluoroscopy-guided transvenous femoral renal transplant biopsy (TFRB) might be a valuable alternative. This study aims to describe the TFRB technique, its safety, and efficacy.

**Methods:**

All patients who underwent TFRB at our institution between 2020 and 2023 were retrospectively analyzed. Procedure outcomes, patients’ characteristics and follow-up data were collected. Adverse events were graded using the Adverse Events Classification of the Society of Interventional Radiology.

**Results:**

From January 2020 to December 2023, 109 patients (median age 61.2 years (Interquartile range (IQR), 54.5–68.6) ; 49 males (45.0%)) underwent 122 TFRB. Transvenous approach was indicated due to high bleeding risk in 61.5% (75/122), limited percutaneous access in 13.9% (17/122), or previous failed PRB in 2.5% (3/122). Biopsy success rate was 94.3%, with 7 graft vein catheterization failures. Adequate Banff 97 criteria specimens were obtained in 68.7% of cases, and a definitive histological diagnosis was achieved in 96.5% of biopsies. Complications occurred in 20.0% of procedures (23/115), including 9 major complications (7.8%), with no significant difference according to the TFRB indication (bleeding risk versus percutaneous limited access) (*p* = 0.70), use of antiplatelet agents (*p* = 0.24) or anticoagulants (*p* = 1.00).

**Conclusion:**

This is the first large cohort study to demonstrates TFRB to be effective and safe in case of contraindication to PRB, providing valuable insights for graft dysfunction management.

**Graphic abstract:**

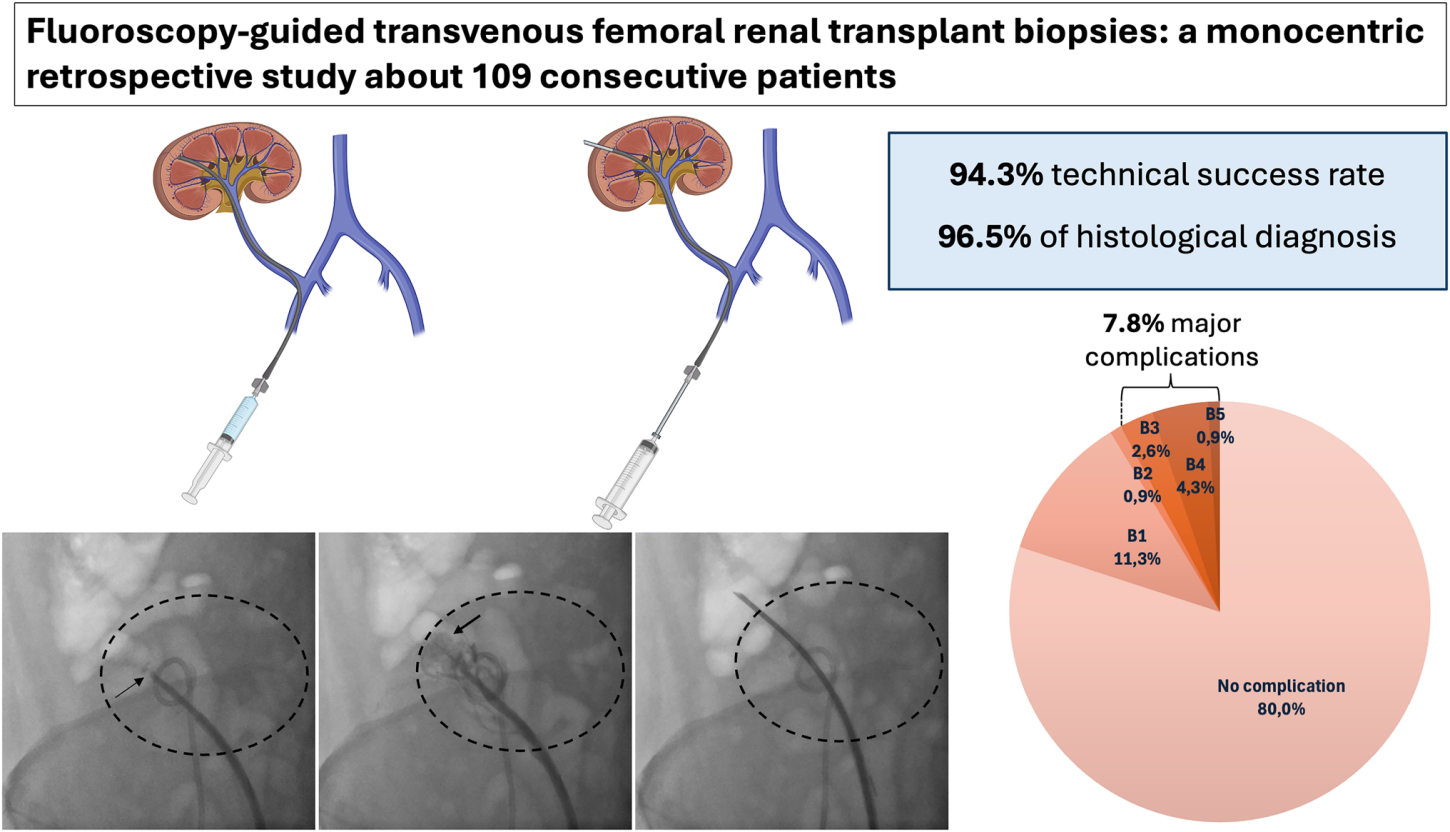

**Supplementary Information:**

The online version contains supplementary material available at 10.1007/s11547-025-02045-4.

## Introduction

Renal transplantation stands out as the most effective treatment for end-stage renal disease in terms of medical outcomes, quality of life, and costs [1]. However, despite significant improvements in immunology, the long-term preservation of allografts remains challenging with approximately 5% of grafts lost annually beyond the initial year [2]. The primary cause of late graft loss is the progression of fibrosis triggered by alloimmune response despite immunosuppressive therapy, followed by cardiovascular risk factors, recurrence of native renal disease, viral infections, and drug toxicity. Anatomopathological analysis is often mandatory for etiological diagnosis of graft dysfunction and subsequent management. Moreover, graft rejection is histologically defined according to the international standardized Banff classification [3].

Percutaneous renal transplant biopsy is generally regarded as safe; however, there are instances where it poses a significant risk. Considered as high-risk bleeding procedure [4], antithrombotic therapy, thrombocytopenia, and other uncorrectable coagulation disorders may be considered as contraindications to percutaneous renal biopsy (PRB). Additionally, limitations to ultrasound-guided biopsy may include difficulty of percutaneous access, especially in cases of obesity, deep grafting, intestinal interpositions, or fluid collection around the graft [5]. To overcome these limitations and contraindications, transjugular venous renal biopsy has been proposed for native renal biopsy [6–10]. Although based on low-level evidence, this technique is recognized as safe and reliable for obtaining histological samples in patients where percutaneous renal biopsy is contraindicated or has failed. In the case of renal transplant patients with contraindications to PRB, transvenous renal transplant biopsy via the ipsilateral femoral vein has been reported [11].

This monocentric retrospective study aims to describe the technique of fluoroscopy-guided transvenous femoral renal transplant biopsy (TFRB), its safety profile and diagnosis efficacy in patients with contraindications to percutaneous biopsy in a large and well characterized population.

## Materials and methods

### Patients

All consecutive patients for which TFRB had been performed in our tertiary nephrology center from January 2020 to December 2023 were retrospectively and independently reviewed by two interventional radiologists (F.T and L.M), with more of 5 years of experience.

Data on patient characteristics, technical data associated with the biopsy and outcomes including adverse events, were collected from the electronic medical record and from the picture archiving and communication system.

The indication for decision to use femoral vein approach rather than percutaneous biopsy was retrospectively collected blindly by a third physician (C.R, with more than 10 years of experience in interventional radiology).

This population has never been previously described.

### Compliance with ethical standards

This retrospective chart review study involving human participants was performed in accordance with the ethical standards of the institutional and national research committee and with the Declaration of Helsinki of the World Medical Association (revised in 2018). The study protocol was reviewed and approved by the Medical Imaging Research Ethics Committee (CRM-2401-389). Written informed consent was obtained from each patient in accordance with the policy of our institution regarding chart reviews. All authors declare that they have no conflicts of interest.

### Procedure description and device

All biopsies were performed by three interventional radiologists (P.C, C.R and E.V) with more than 10 years’ experience in the Advanced Interventional Radiology Department of Pitié-Salpêtrière University Hospital (Paris, France). For each patient, coagulation tests (Platelet count, PT and aPTT ratios) were obtained and checked before biopsy.

All procedures were performed under fluoroscopy in an Artis Zee angiography suite (Siemens Healthcare, Chalfont St. Giles) with local anesthesia (Xylocaïne 1% + Bicarbonate 8.4%) after sterile preparation and aseptic conditions.

Punction of the ipsilateral femoral vein was systematically performed under ultrasound guidance (RS85, Samsung).

After introduction of a 5 French (F) introducer sheath (Radiofocus®, Terumo), renal transplant vein was catheterized with a 5-F catheter, either RC1 (Torcon NB® Advantage catheter, Cook) or Cobra 2 (Radiofocus®, Terumo), and a curved hydrophilic guide 0.035-inch (Radiofocus®, Terumo).

After reaching peripherical vein as distally as possible at the upper pole of the transplant, the hydrophytic guidewire was exchanged for a metallic guidewire (Amplatz Super Stiff guidewire, Boston Scientific®). The 5-F sheath was then removed and replaced by a 9-F armed 24 cm sheath (Arrow®).

**Figure 1:**
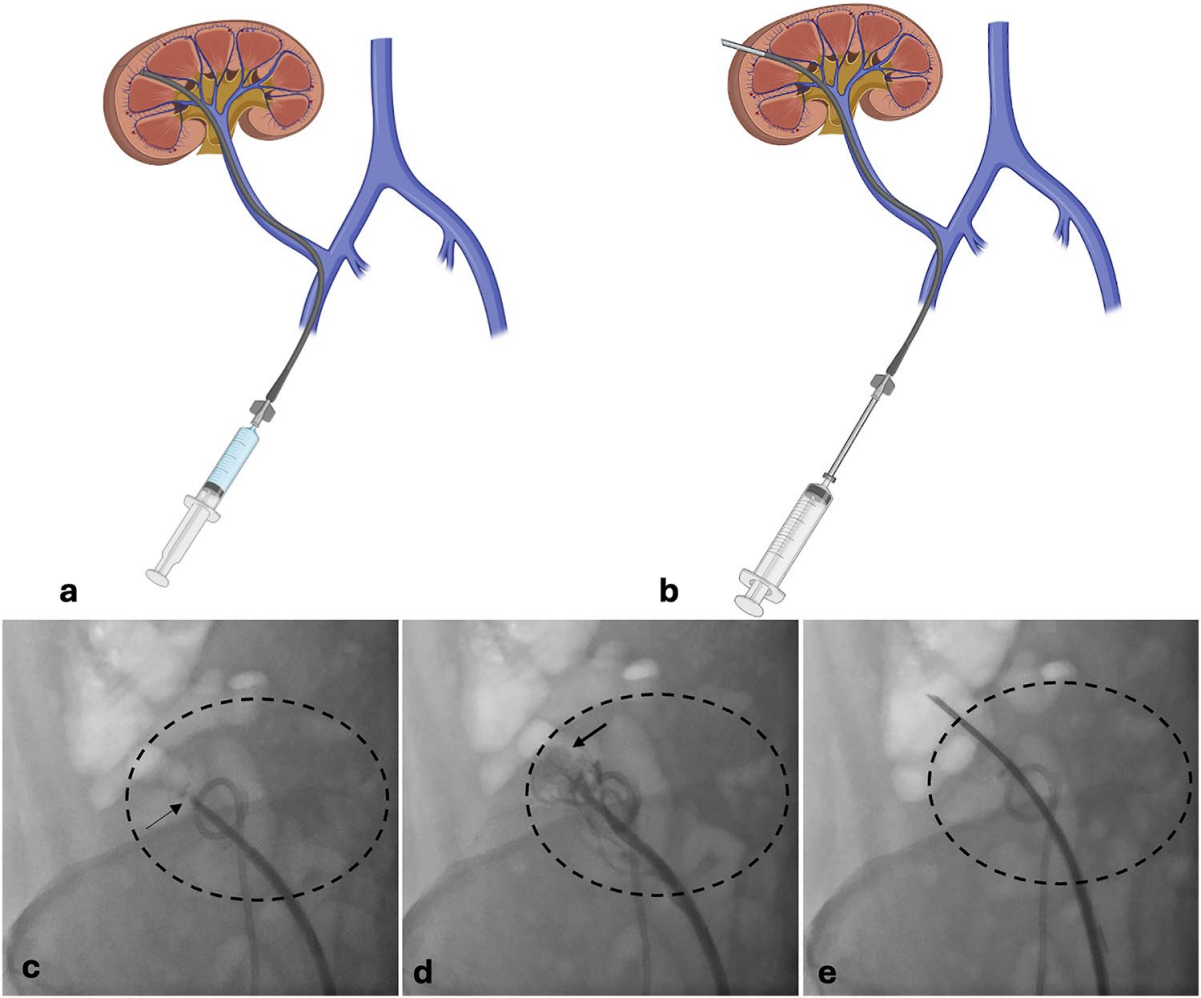
Fluoroscopy-guided transvenous femoral renal transplant biopsy. Schematic (**a**) and angiographic (**c**) representation of distal catheterization of the renal transplant vein (thin arrow), verification of correct positioning by injection of iodinated contrast medium (**d**) showing the cortical wedge nephrography (thick arrow) and biopsy needle sampling (**b** and **e**).

**Figure 2:**
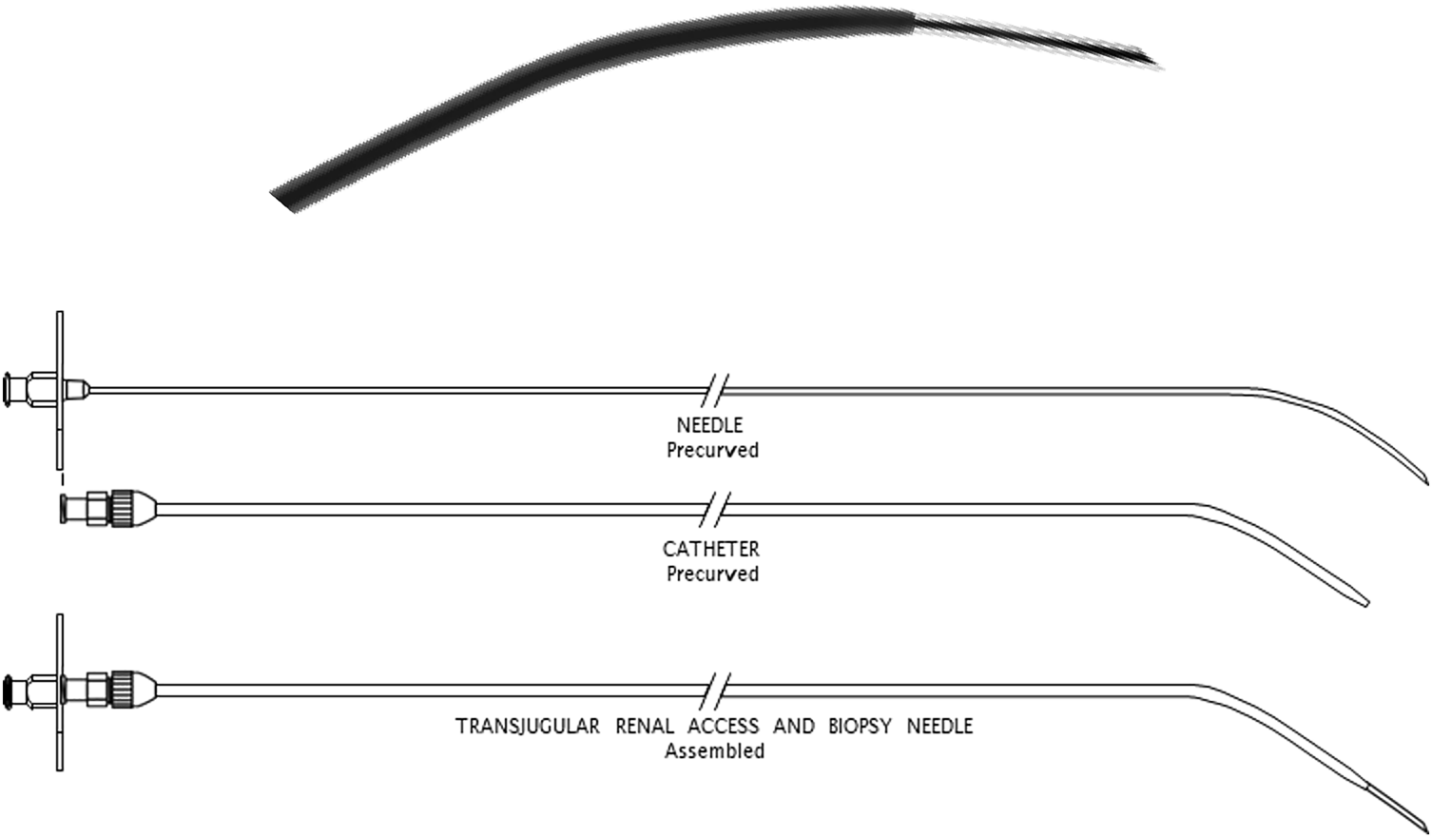
Transjugular Renal Access and Biopsy Needle (Cook®).

The precurved catheter of Transjugular Renal Access (Cook®) is brought distally to the superior pole of the transplant (Figure 1a and c) and correct positioning of the sheath is verified by a gentle injection of diluted contrast medium (Iodixanol 320 mg iodine/ml, GE Healthcare), revealing a focal retrograde cortical wedge nephrography (Figure 1d). The biopsy is then performed using a 64 cm length 15-Gauge (G) Biopsy Needle (Cook®) (Figure 2). The needle, after been oriented laterally to avoid accidental intestinal punction, is pushed into the parenchyma taking care to cross the renal capsule (Figure 1b and e) and then removed in one continuous movement while maintaining suction with a 20 mL syringe.

**Figure 3:**
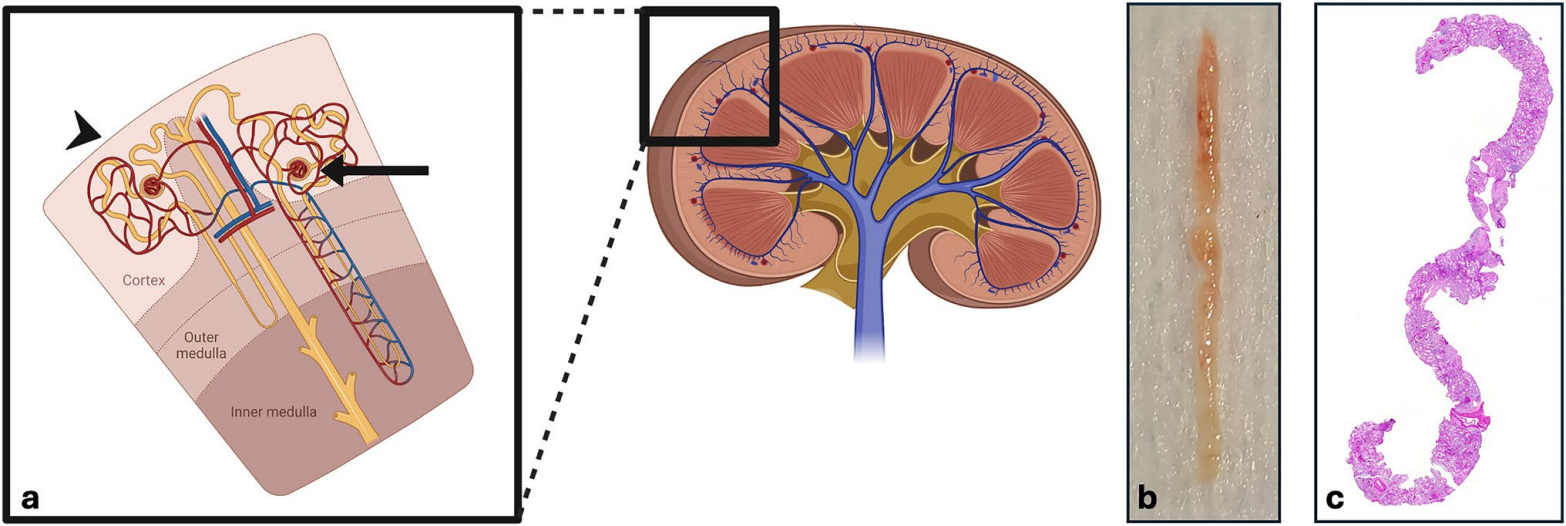
Representative images of glomeruli distribution on a schematic anatomical illustration of the kidney **a** highlighting the presence of glomeruli in the renal cortex and in a biopsy sample in macroscopic **b** and microscopic **c** views.

This operation was performed twice to obtain two samples, one directly fixed in Formol acetic alcohol for light microscopy and another applied to a compress hydrated with physiological serum for immunofluorescence microscopy on frozen tissue. Samples’ length of more than 1 cm and glomeruli presence macroscopically assessed defined the technical success of the procedure (Figure 3a and b). If at least two samples were not considered to fulfill both those criteria, a third pass was performed. At the end of the procedure, a soft compression of the femoral vein is performed after removal of devices. No systemic therapy was administrated during the procedure.

### Pathologic analysis

Samples were analyzed by the nephropathologist specialized at our institution. Light microscopy was performed on fixed samples and stained with HES, PAS, Jones silver stain, and trichrome. Immunofluorescence was performed on fresh samples. If needed, electron microscopy was performed on fixed samples, using glutaraldehyde as fixative solution. Specimen adequacy was determined based on the Banff classification criteria: minimal sample of seven glomeruli and one artery and adequate sample of 10 glomeruli and two arteries [12].

### Follow-up and complications

Patients were monitored for at least 2 h in the recovery room, with assessment of urine appearance and pain. In event of unusual pain or significant macroscopic hematuria, an ultrasound or CT scan was performed looking for early post-biopsy complication. All patients underwent at least 12 h of strict decubitus after biopsy to prevent complications and were hospitalized for at least 24 h. Hemoglobin and serum creatinine were reassessed within 48 h of biopsy. Contrast-induced nephropathy was defined as a 25% increase in serum creatinine over pre-biopsy levels within 3 days of contrast administration. After patients were discharged, they returned to routine follow-up.

Complications were graded according to the New Adverse Event Classification proposed by the Society of Interventional Radiology [13].

### Statistical analysis

All statistical tests were two-tailed, and *p* values < 0.05 were considered statistically significant. Categorical variables, presented as numbers with percentages, were analyzed with the χ2 test or Fisher’s exact test, depending on the number of patients. Continuous variables, presented as median and interquartile range (IQR, Q1–Q3) or minimum and maximum values (Min–Max), were analyzed using the Mann–Whitney U test. Paired data were analyzed using the Wilcoxon or McNemar signed ranks test, as appropriate. Statistical analysis was performed with the statistics software “R” (version 2023.09.1+494).

## Results

### Patients’ characteristics

Between January 2020 and December 2023, 122 consecutive TFRB from 109 patients were performed in our institution (60 females (55.0%); 49 males (45.0%)), with a median age of 61.2 years (IQR, 54.5–68.6). Patients and transplants characteristics are presented in Table 1 and indications for biopsy and transvenous approach in Table 2.

**Table 1:** Patients and transplants characteristics

*Patients characteristics*	*n = 109*
Male, n (%)	49 (45.0)
Age, years, median (IQR)	61.2 (54.5–68.6)
In-patients, n (%)	64 (58.7)
*Initial nephropathy, n (%)*	
Indeterminate	21 (19.3)
Diabetes	16 (14.7)
ADPKD	12 (11.0)
NAS	11 (10.1)
FSHS	10 (9.1)
Vascular origin	8 (7.3)
IgAN	4 (3.7)
APS	6 (5.5)
Other^a^	21 (19.3)

*Transplants characteristics*	*n = 109*
*Transplant laterality, n (%)*	
Right iliac fossa	94 (86.2)
Left iliac fossa	15 (13.8)
*Number of transplant, n (%)*	
First	95 (87.2)
Second	12 (11.0)
Third	2 (1.8)
*Type of transplant, n (%)*	
Kidney alone	101 (92.6)
Kidney and liver	4 (3.7)
Kidney and heart	4 (3.7)

^a^*Other initial nephropathy : hydronephrosis, HIV-associated nephropathy, amyloidosis, lithiasis, Alport syndrome, vasculitis*

*ADPKD, autosomal dominant polycystic kidney disease; NAS, Nephroangiosclerosis ; FSHS, focal and segmental hyalinosis and sclerosis ; IgAN, IgA nephropathy ; APS, antiphospholipid syndrome*

**Table 2:** Indications for biopsy and transvenous approach

*Transplant biopsy*	*n = 122*
Time from transplant to biopsy, months, median (IQR)	26.0 (10.5–72.0)
Within one month, n (%)	5 (4.1)
*Biopsy indication, n (%)*	
Progressive dysfunction	29 (23.8)
Acute dysfunction	26 (21.3)
Proteinuria	21 (17.2)
DSAdn	18 (14.8)
Protocol	12 (9.8)
Rejection follow-up	9 (7.4)
Suspected BK virus infection	4 (3.3)
Other^a^	3 (2.5)
*Pre-biopsy coagulation tests, median (IQR)*	
Platelets, 10^9^/L	206.0 (167.0–256.5)
PT ratio, %	94.0 (85.0–100.0)
aPTT ratio	0.99 (0.92–1.09)

*Transvenous approach indication*	*n = 122*
*Hemostasis disorders, n (%)*	*75 (61.5)*
Curative anticoagulation	30 (24.6)
Single antiplatelet therapy	38 (31.1)
Aspirin only	37 (30.3)
Clopidogrel only	1 (0.8)
Dual antiplatelet therapy	2 (1.6)
Thrombocytopenia	3 (2.5)
Coagulation disorder	2 (1.6)
*Limited percutaneous access, n (%)*	*17 (13.9)*
Obesity	6 (4.9)
Peri-graft collection (lymphocele or hematoma)	6 (4.9)
Digestive tract interposition	4 (3.3)
Deep graft	1 (0.8)
*Percutaneous biopsy failure, n (%)*	*3 (2.5)*
*Other*^b^*, n (%)*	*27 (22.1)*

^a^*Other biopsy indication : bartonellosis, thrombotic microangiopathy, amyloidosis*

^***b***^*Other transvenous approach indication : arteriovenous fistula, unknown*

*DSAdn, de novo donor specific antibodies ; PT, prothrombin time ; aPTT, activated partial thromboplastin time*

The allograft was in the left iliac fossa in 15 patients (13.8%). The median time from transplant to biopsy was 26.0 months (IQR, 10.5–72.0), and five procedures (4.1%) were attempted within 1 month of transplant.

The transvenous approach was considered because of the high bleeding risk in 75 cases of 122 (61.5%). Of these, 30 (24.6%) for curative anticoagulation, 2 (1.6%) for dual antiplatelet therapy, 38 (31.1%) for single antiplatelet therapy, and 5 (4.1%) for coagulopathy and thrombocytopenia. Further details on antiplatelet and anticoagulant treatments and their indications are provided in Supplementary Tables 1 and 2. In 17 cases of 122 (13.9%), TFRB was indicated because of limited percutaneous access due to obesity, peri-graft collection (lymphocele or hematoma), digestive tract interposition, or deep graft. Finally, transvenous indication was previous failed PRB for three cases of 122 (2.5%) or other indication as arteriovenous fistula on previous biopsy.

All patients underwent laboratory tests before biopsy presented in Table 2. Thirteen patients (10.7%) had aPTT ratio < 1.2 and 1 patient (0.8%) had PT ratio < 50% but all patients had platelet count > 50 10^9^/L.

### Procedure and samples’ characteristics

Procedure technical success and outcomes are presented in Table 3. Transvenous transplant biopsy was achieved in 115 of 122 procedures (94.3%). In seven cases, angulation of the graft vein anastomosis prevented venous catheterization.

**Table 3:** Procedure technical outcomes and samples characteristics

*Procedure outcomes*	*n = 122*
Duration, minutes, median (IQR)	35.8 (28.0–43.6)
Fluoroscopy time, minutes, median (IQR)	3.1 (2.1–4.8)
Radiation dose, µGy.m^2^, median (IQR)	906.9 (476.9–2157.8)
Number of passes, median (Min-Max)	2 (0–5)
Number of biopsy cores, median (Min-Max)	2 (0–4)
Failed vein graft catheterization, n (%)	7 (5.7)

*Samples characteristics*	*n = 115*
Adequate tissue for diagnosis^a^, n (%)	79 (68.7)
Length of core samples, mm, median (IQR)	10.0 (7.0–12.3)
Glomeruli number, median (IQR)	13.5 (8.7–18.2)
Over 10 glomeruli, n (%)	75 (65.2)

^a^*According to the Banff criteria used at the time of the biopsy.*

The median number of passes was 2 (Min–Max, 0–5) yielding two biopsy cores (Min–Max, 0–4). Median procedure duration was 35.8 minutes (IQR, 28.0–43.6), fluoroscopy time was 3.1 min (IQR, 2.1–4.8) and radiation dose was 906.9 µGym^2^ (IQR, 476.9–2157.8).

According to the Banff criteria used at the time of the biopsy, tissue sample was adequate for diagnosis for 79 of 115 biopsies (68.7%) but it permitted definitive histological diagnosis in 111 of 115 (96.5%). Median length of core samples was 10.0 mm (IQR, 7.0–12.3) with a median of 13.5 glomeruli per biopsy (IQR, 8.7–18.2). Out of 75 samples (65.2%), more than 10 glomeruli were available for analysis.

### Outcome and complications

The median time to discharge from hospital after biopsy was 1 day (IQR, 1.0–2.0). Among the 98 biopsies out of 115 for which the post-biopsy biological test results were available, 7 patients (7.1%) had a more than 25% increase in post-biopsy serum creatinine compared with pre-biopsy serum creatinine (Table 4).

**Table 4:** Pre- and post-biopsy laboratory tests

	*Pre-biopsy (n = 122)*	Post-biopsy *(n = 115)*^*a*^	*p*
Hemoglobin, g/dL, median (IQR)	11.0 (9.7–12.1)	10.7 (9.5–11.7)	*0.052*
Creatinine, µmol/L, median (IQR)	201.5 (163.0–250.2)	188.0 (152.0–259.0)	*0.854*
Post-biopsy increase > 25%^b^, n (%)		7 (7.1)	

^a^*Post-biopsy biological tests were not available for 17 patients.*

^b^*> 25% increase in post-biopsy serum creatinine compared with pre-biopsy serum creatinine within 3 days.*

**Figure 4:**
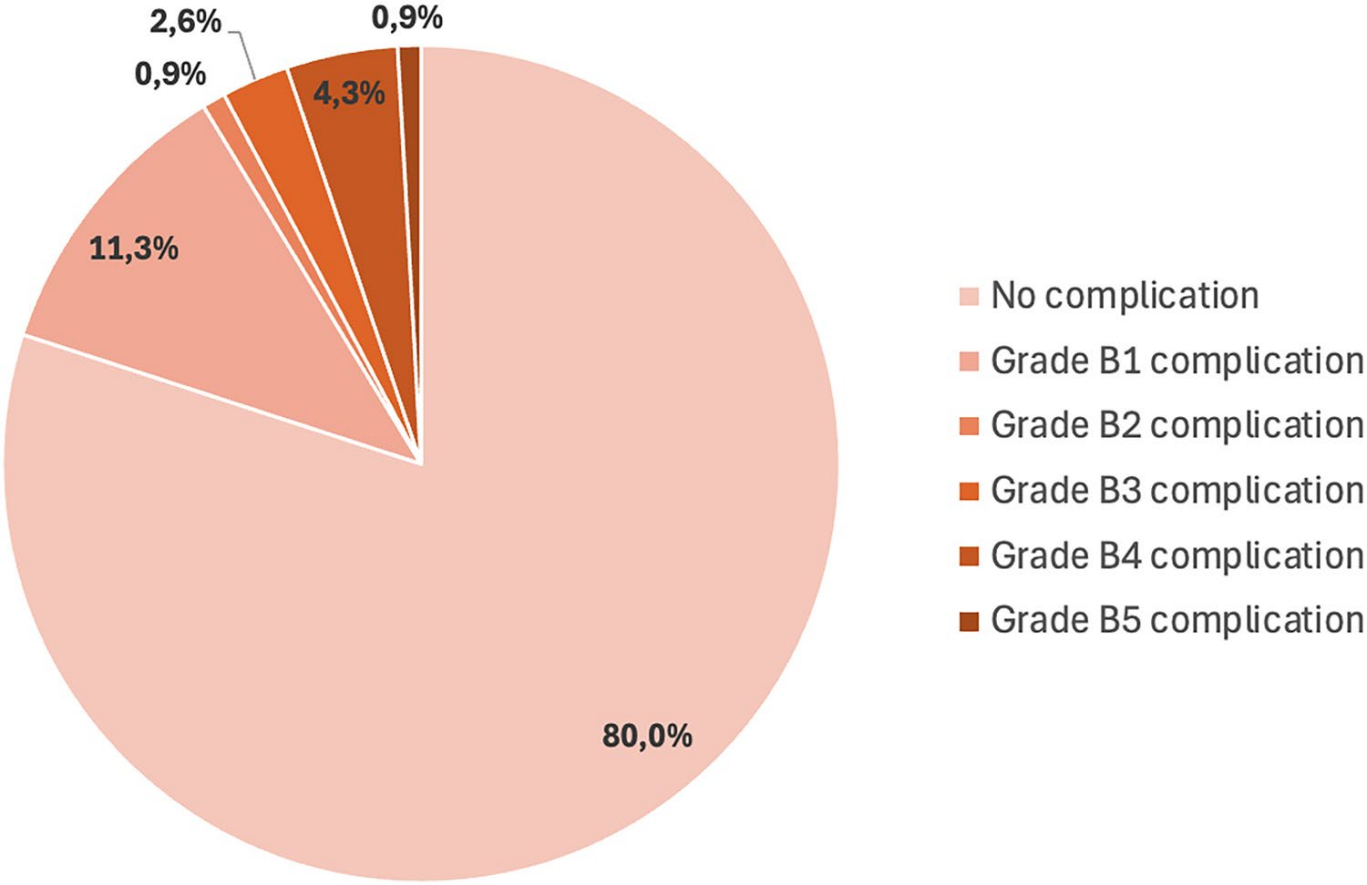
Grade distribution of complications according to Adverse Events Classification of the Society of Interventional Radiology [13].

We observed 23 complications out of 115 procedures (20.0%) presented in Table 5 and Figure 4, including 9 (7.8%) major complications (i.e., ≥ grade B3). All were bleeding complications, except for one septic shock.

**Table 5:** Classification and therapeutic management of complications

*Classification of complications*^*a*^*, n (%)*	*n = 115*^*b*^
*All*	*23 (20.0)*
*Mild B1*	*13 (11.3)*
Self-limiting gross hematuria	9 (7.8)
Bladder catheter	4 (3.5)
*Moderate B2*	*1 (0.9)*
Blood transfusion	1 (0.9)
*Severe B3*	*3 (2.6)*
Double J ureteral catheter	2 (1.7)
48-hour extended hospital stay	1 (0.9)
*Life-threatening B4*	*5 (4.3)*
Hemorrhagic shock	4 (3.5)
Septic shock	1 (0.9)
*Death B5*	*1 (0.9)*

*Therapeutic management, n (%)*	
Spontaneous improvement	9 (39.1)
Continuous bladder irrigation	10 (43.5)
Double J ureteral catheter	2 (8.7)
Blood transfusion	6 (26.1)
Use of vasopressors	5 (21.7)
Arterial embolization	3 (11.1)

^a^*Complications severity graded according to the New Adverse Event Classification proposed by the Society of Interventional Radiology* [13]* (grade based on most serious complication).*

^b^*All procedures excepted the seven failed vein graft catheterization*

Among non-major complications, 9 patients (7.8%) presented a self-limiting gross hematuria, 4 (3.5%) required bladder catheterization (mild complication B1), and 1 (0.9%) required a blood transfusion (moderate complication B2).

**Figure 5:**
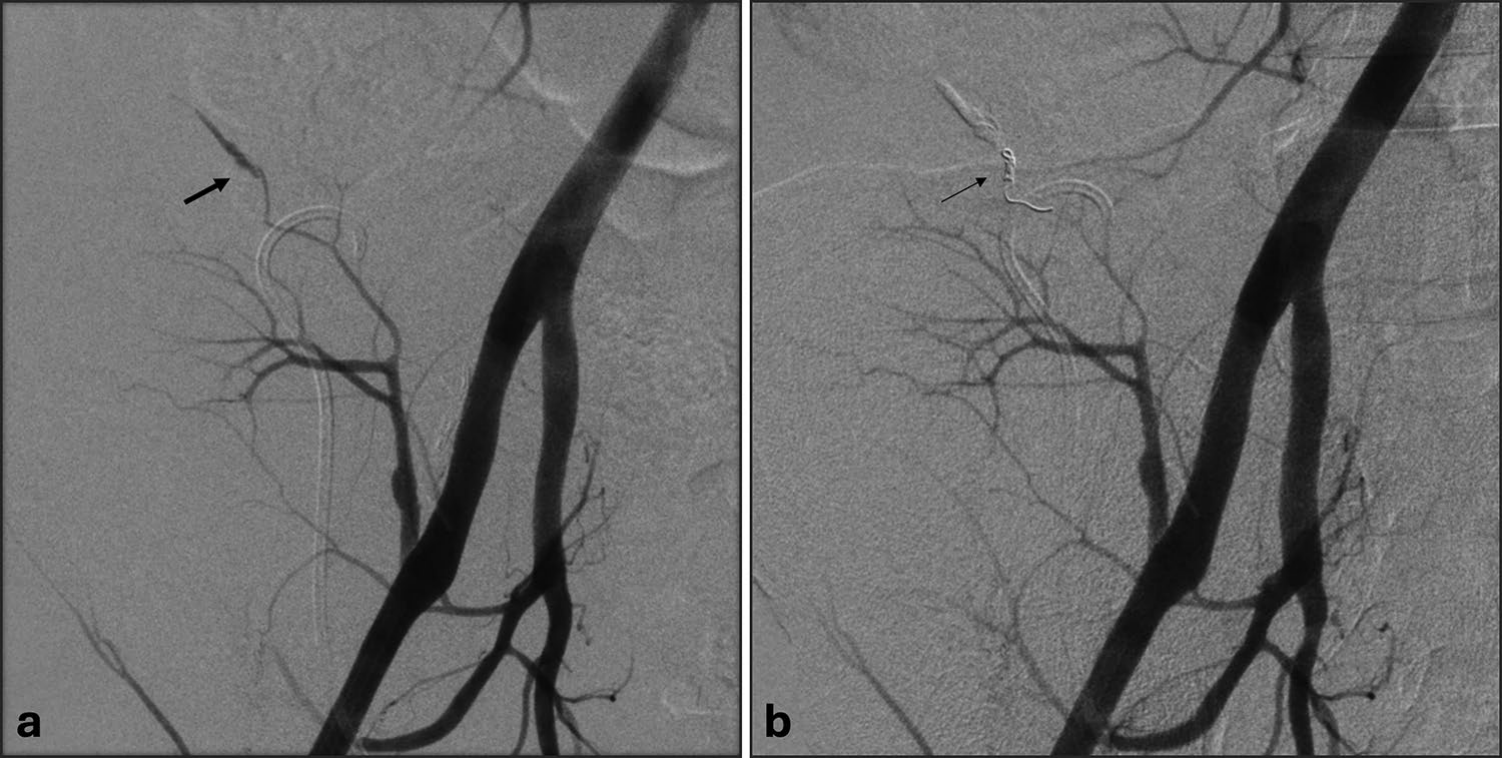
Representative case of post-biopsy arterial bleeding managed by coil embolization. Angiographic images of active bleeding (thick arrow) from an arterial branch of the renal transplant **a** and control angiography **b** after embolization with coils (thin arrow).

Three patients presented a severe grade B3 complication (2.6%), two required ureteral catheterization, and one patient was hospitalized for 48 h after biopsy. Five patients presented a life-threatening B4 complication (4.3%). Of these, four suffered hemorrhagic shock, of which three requiring transarterial embolization, and one septic shock treated with appropriate antibiotics. Figure 5 illustrates a case of post-biopsy arterial bleeding from a branch of the renal transplant, successfully treated by endovascular coil embolization performed within 4 h after the biopsy.

The patients with major bleeding underwent CT scan within 3 h and 8 days after the biopsy. One patient (0.9%) died of acute respiratory failure following hemorrhagic shock (grade B5 complication) immediately after the CT scan. Embolization was performed on the remaining three patients, within 30 min and 2 h after the diagnosis.

Renal function remained stable in every patients after arterial embolization at hospital discharge. None of these patients required surgery.

More details on the four patients who suffered hemorrhagic shock regarding the pre-biopsy risk of bleeding are presented in Supplementary Table 3.

Regarding to the occurrence of severe hemorrhagic complications (i.e., ≥ grade B3), there was no significant difference according to the use of antiplatelet and anticoagulant or not (*p* = 0.24 and *p* = 1.00, respectively), or according to the indication due to a high bleeding risk or limited percutaneous access (*p* = 0.70).

## Discussion

Fluoroscopy-guided transvenous femoral renal transplant biopsy remains a rarely described technique in the literature, particularly regarding its technical aspects, diagnostic yield, and safety profile. To date, only one small retrospective study has been published [11].

Our study is the first large cohort investigating this innovative approach. We demonstrated a high feasibility (94.3% technical success rate) and effectiveness of this technique (96.5% histological diagnosis established) with an acceptable complication rate (7.8% of major complications) observed in a high-risk patient population. Additionally, the bleeding risk did not differ significantly between patients treated with anticoagulant or antiplatelet therapies and those with limited percutaneous access.

The biopsy success rates were high, with only 5.7% experiencing graft vein catheterization failures, and a definitive histological diagnosis achieved in 96.5% of cases. This success rate slightly exceeds that reported by Schmid et al., who achieved a completion rate of 81.7% [11]. In addition, we obtained a 68.7% rate of adequate samples according to the Banff criteria, compared to 50.9% in the study by Schmid et al. These figures are in line with published data for percutaneous biopsies, where adequacy rates range from 55% to 85% [14–16].

Several factors may explain the favorable diagnostic yield of our study. We used a larger 15-G needle, while Schmid et al. used a 19-G needle. The advantage of using larger needle sizes (14-G or 16-G) in renal transplant biopsies has been demonstrated in a prospective randomized trial [17]. Furthermore, the consistent crossing of the renal capsule during sampling, a technique adopted in our center, is associated with improved core quality [18].

In our cohort, the rates of minor and major complications following TFRB were 12.2% and 7.8%, respectively. Although these rates appear higher than those reported for ultrasound-guided percutaneous transplant biopsies, (minor : 0–4% ; major : 3–8%) [14, 19–22], it is important to note that 61.5% of our study’s patients had an increased bleeding risk due to antithrombotic therapy or hemostasis disorders. These patients are typically excluded from percutaneous graft biopsy studies, making direct comparison challenging. Consequently, our complication rates are closer to those seen in transjugular biopsies of native kidneys (18.2% minor, 4.5% major complications) [5]. Another factor influencing complication risk is the needle size used, with lower risks associated with needles of 18 G or more [21].

In our study, the use of antiplatelet or anticoagulants was not associated with a higher risk of serious hemorrhage compared to patients undergoing biopsy for limited percutaneous access. This finding tends to confirm the hypothesis that transvenous biopsies are associated with lower risk of bleeding and are the safest way to obtain biopsies in patients with high bleeding risk, consistent with earlier observations by Cluzel et al [6]. In patients on anticoagulation therapy, avoiding bridging strategies, which increase bleeding risks, further reinforces the value of TFRB. Moreover, for patients requiring dual or single antiplatelet therapy, TFRB allows biopsy without interrupting treatment, an important consideration in populations with high cardiovascular risk [23].

TRFB offers the advantage of obtaining a histological sample when percutaneous biopsies are contraindicated. However, it also presents logistical drawbacks, including higher costs of consumables and longer procedure duration. In a recent study, percutaneous renal biopsy had a mean procedure time of 20.6 minutes [16], compared to 35.8 minutes for TFRB in our study. Additionally, TFRB requires the use of X-rays for endovascular guidance, although radiation levels remain reasonable within standard radiation protection rules and with a median fluoroscopic time of 3 min in our study.

Beyond logistical considerations, TFRB carries a risk of contrast-induced nephropathy, although low. We observed a significant increase in serum creatinine after TFRB in 7.1% of cases. These rates are comparable to those reported in transvenous biopsy of native kidney and graft, of 7.9% and 5.7%, respectively [9, 11]. Given the context of underlying graft dysfunction, distinguishing contrast-induced nephropathy from disease progression remains difficult.

The technical complexity of this procedure and the absence of a dedicated device present additional potential obstacles to the widespread of TFRB. In our study, we used a large-bore needle to obtain adequately sized samples, which also increases the risk of bleeding complications. The use of a metallic guidewire (e.g., Amplatz Super Stiff guidewire), as described in the procedure, facilitates needle passage by opening the angle of the graft vein. In addition, it is crucial to perforate the graft capsule to obtain better samples and limit the risk of subcapsular hematoma. Given these considerations, this technique should be reserved for experienced, high-volume, multidisciplinary tertiary centers because with these precautions in mind, and despite these drawbacks, TFRB is feasible, safe and effective.

Although our study presents the largest study population on this subject in the published literature, its main limitation lies in its non-comparative design, making it difficult to compare our study population with patients without contraindications for percutaneous renal biopsies. While the rate of serious complications appears acceptable, a prospective, comparative study would be necessary to clarify the place of TFRB in the diagnostic field for suspected graft rejection with contraindication to PRB.

In conclusion, fluoroscopy-guided transvenous renal transplant biopsy has demonstrated feasibility along with good diagnostic performances. When no other option is available, TFRB enables histological diagnosis and thus patient management with a good safety profile, even though it is aimed at a fragile population.

## Fundings

The authors declare that no funds, grants, or other support were received during the preparation of this manuscript.

## Supplementary Information

Below is the link to the electronic supplementary material.

Supplementary file1 (DOCX 17 KB)

## Abbreviations

ADPKD: Autosomal Dominant Polycystic Kidney Disease
APS: Antiphospholipid Syndrome
aPTT: Activated Partial Thromboplastin Time
DOACs: Direct Oral Anticoagulants
DSAdn: de novo Donor Specific Antibodies
FSHS: Focal and Segmental Hyalinosis and Sclerosis
Hb: Hemoglobin
IgAN: IgA Nephropathy
IQR: Interquartile Range
NAS: Nephroangiosclerosis
OALL: Obliterating Arteriopathy of the Lower Limbs
PRB: Percutaneous Renal Biopsy
PT: Prothrombin Time
TFRB: Transvenous Femoral Renal transplant Biopsy
VKA: Vitamin K Antagonist
