# Supplementary material for: Fluoroscopy-guided transvenous femoral renal transplant biopsies: a monocentric retrospective study about 109 consecutive patients
Source: Radiol Med. 2025 Aug 2;130(10):1711–21. doi: 10.1007/s11547-025-02045-4 (PMC12546500; doi:10.1007/s11547-025-02045-4)
Supplement: Supplementary file 1 — Supplementary file1 (DOCX 17 KB) [file 11547_2025_2045_MOESM1_ESM.docx]

Supplementary Table 1 : Type of antiplatelet drugs and indications

| ***Type*** | *n = 40* |
| --- | --- |
| Aspirin only, n (%) | 37 (92.5) |
| Clopidogrel only, n (%) | 1 (2.5) |
| Dual antiplatelets, n (%) | 2 (5.0) |
| ***Indication*** | *n = 40* |
| Primary prevention, n (%) | 12 (30.0) |
| Non-stented coronary artery disease, n (%) | 7 (17.5) |
| Stented coronary artery disease, n (%) | 11 (27.5) |
| Stroke, n (%) | 3 (7.5) |
| Not specified, n (%) | 7 (17.5) |

Supplementary Table 2 : Type of anticoagulant and indications

| ***Type*** | *n = 30* |
| --- | --- |
| Heparin, n (%) | 12 (40.0) |
| DOACs, n (%) | 11 (36.7) |
| VKA, n (%) | 6 (20.0) |
| Danaparoid sodium, n (%) | 1 (3.3) |
| ***Indication*** | *n = 30* |
| Atrial fibrillation, n (%) | 15 (50.0) |
| Venous thromboembolism, n (%) | 9 (30.0) |
| APS, n (%) | 1 (3.3) |
| OALL, n (%) | 1 (3.3) |
| Not specified, n (%) | 4 (13.3) |
| *DOACs, direct oral anticoagulants ; VKA, vitamin K antagonist ; APS, antiphospholipid syndrome ; OALL, obliterating arteriopathy of the lower limbs* | |

Supplementary Table 3 : Data on the four patients with hemorrhagic shock

| ***Patient’s condition*** | ***Bleeding risk*** | ***Pre-biopsy creatinine (µmol/L)*** | ***Pre-biopsy hemoglobin (g/dL)*** | ***Evolution*** |
| --- | --- | --- | --- | --- |
| Viral liver disease | Thrombocytopenia (platelet count 66.10^9^/L) | 188 | 13.4 | Cardiorespiratory arrest within 6 hours |
| Recent history of pulmonary embolism | Bridging anticoagulation therapy with heparin | 457 | 7.9 | Successful arterial embolization |
| Embolism cardiopathy | Bridging anticoagulation therapy with heparin | 208 | 10.1 | Successful arterial embolization |
| Double heart-kidney transplantation | Bridging anticoagulation therapy with heparin | 491 | 8.4 | Successful arterial embolization |
